# Pan-Cancer Analysis of Potential Synthetic Lethal Drug Targets Specific to Alterations in DNA Damage Response

**DOI:** 10.3389/fonc.2019.01136

**Published:** 2019-10-25

**Authors:** Shaoli Das, Kevin Camphausen, Uma Shankavaram

**Affiliations:** Radiation Oncology Branch, National Cancer Institute, National Institutes of Health, Bethesda, MD, United States

**Keywords:** DNA damage response, synthetic lethality, single sample gene set enrichment analysis, mutual exclusivity, somatic mutations, drug sensitivity, disease-free survival, Kaplan-Meier analysis

## Abstract

Alterations in DNA damage response (DDR) is one of the several hallmarks of cancer. Genomic instability resulting from a disrupted DDR mechanism is known to contribute to cancer progression, and are subjected to radiation, cytotoxic, or more recently targeted therapies with limited success. Synthetic lethality (SL), which is a condition where simultaneous loss-of-function of the genes from complementary pathways result in loss of viability of cancer cells have been exploited to treat malignancies resulting from defects in certain DDR pathways. Albeit being a promising therapeutic strategy, number of SL based drugs currently in clinical trial is limited. In this work we performed a comprehensive pan-cancer analysis of alterations in 10 DDR pathways with different components of DNA repair. Using unsupervised clustering of single sample enrichment of these pathways in 7,272 tumor samples from 17 tumor types from TCGA, we identified three prominent clusters, each associated with specific DDR mechanisms. Somatic mutations in key DDR genes were found to be dominant in each of these three clusters with distinct DDR component. Using a machine-learning based algorithm we predicted SL partners specific to somatic mutations in key genes representing each of the three DDR clusters and identified potential druggable targets. We explored the potential FDA-approved drugs for targeting the predicted SL genes and tested the sensitivity using the drug screening data in cell lines with mutation in the primary DDR genes. We have shown clinical relevance, for selected targetable SL interactions using Kaplan-Meier analysis in terms of improved disease-free survival. Thus, our computational framework provides a basis for clinically relevant and actionable SL based drug targets specific to alterations in DDR pathways.

## Introduction

Responding to DNA damage from various internal or external stimuli is a crucial process for cell viability. Normally the DNA repair pathways guide the cell fate decisions for cells exposed to DNA damage; they can either be repaired and restored to normal function, or in cases where the damage is irreversible the cell is “sacrificed” by senescence via activation of certain DNA damage response (DDR) pathways (1). But in a third scenario, because of inefficient repair of damaged DNA, the affected cell evades senescence, which leads to proliferation of cells carrying oncogenic alterations, and subsequently develops cancer (2). Genomic instability caused by driver mutations is a hallmark of cancer (3). A key function of the DDR machinery in cancer cells is to promote genomic stability and guiding cell fate decisions. Traditional cancer therapies involving radiation and cytotoxic chemotherapies induce DNA damage and exploit DDR pathways to facilitate tumor cell death. Thus, DDR pathways play a major role to determine response to these therapies (4).

DNA damage response (DDR) is a complex multi-level process involving sub-pathways like base-excision, nucleotide excision and mismatch repair for handling single-strand breaks, or homologous recombination repair, homology directed repair, non-homologous end joining, and Fanconi anemia pathways for handling double-strand breaks in DNA (5). Deficiency and alterations in various components of the DDR machinery is common in all types of cancers (6), but generally deficiency in one component can be compensated by other components of DDR (2, 7). Synthetic lethal (SL) interactions are formed between components which are compensatory between themselves. So, a better understanding of the SL relationships between components of DDR is a promising approach to tackle resistance to conventional cancer therapy. Guided by the knowledge of specific DDR alterations in individual patients, SL-based drug targets can be attractive choices for personalized cancer therapy.

The success of *PARP* inhibitors in treating *BRCA1/2* mutated tumors in clinical trials demonstrated the validity of the concept of SL (8). FDA approval of *PARP* inhibitor drugs for treating *BRCA1/2*-mutated ovarian and breast cancer patients has propelled interest in exploration of other potential SL associations between DDR components. Use of shRNA or CRISPR screenings in cancer cell lines is a viable approach for identifying synthetic lethal interactions specific to certain cancer genes of interest (9, 10), but running these screenings are costly when the number of the genes of interest is large. On the other hand, computational prediction of cancer-specific SL interactions can identify many potential candidates for SL interactions (11, 12), but without proper validation of these predictions it is hard to prioritize the targets. We tried to address this limitation by our previously published machine-learning based computational method called DiscoverSL that harnesses the large-scale tumor genomic and clinical data from cancer patients combined with the RNAi and drug screening data from cancer cell lines to infer statistical measures on predicted synthetic lethal interactions to prioritize clinically relevant and targetable candidates (13). Another computational method ISLE also prioritizes SL pairs by identifying those pairs that are predictive of patients' survival upon co-inactivation; but they use literature-derived SL interactions from shRNA-screening experiments (14). Driven by the need to identify potential SL based drug targets specific to alterations in certain DDR pathways in cancers, here we performed a pan-cancer analysis on enrichments or deficiencies of different DDR pathways and alterations in the DDR components from genomic data of 17 tumor types from The Cancer Genome Atlas (TCGA). Combining the existing knowledgebase on potential SL interactions from literature and the SL predictions from DiscoverSL algorithm with the FDA-approved drug targets, we propose clinically relevant, and potentially actionable, cancer specific network of SL DDR alterations, and drug interactions.

## Materials and Methods

### Data Source and Pre-processing

The primary source of tumor genomic and clinical data of 17 tumor types is the cancer genome atlas (TCGA) project (15). Somatic mutation, and RSEM processed and Z-score normalized RNA-Seq v2 gene expression data of TCGA tumor samples are downloaded from cBioPortal (16). Additionally, raw RNA-Seq count data of TCGA tumor, and normal samples was collected from a published resource from Gene Expression Omnibus accession GSE62944, that processed TCGA raw RNA-Seq data using featurecount package to generate the gene-wise raw counts (17).

The gene-pathway associations for 10 DDR pathways were collected from the curated geneset (c2 version 6.2) in the MsigDB database (18).

For validation purposes, we collected processed shRNA screening data in 214 cancer cell lines from Achilles project version 2.4.3 in form of essentiality scores calculated using ATARiS (19). Genomic profiles (mutation) of these cancer cell lines are collected from the cancer cell line encyclopedia [CCLE (20)].

Drug-protein interaction data are collected from the databases DrugBank and DGIDB (21, 22). For drug sensitivity analysis we collected drug screening data from the genomics of drug sensitivity in cancer (GDSC) data portal (23). From this portal we collected the drug response data in cancer cell lines using LN-IC50 and AUC scores as well as the genomic mutation profiles of the corresponding cancer cells.

### Computational and Meta-Data

Single sample enrichment scores for 10 DDR pathways across 7,272 tumor samples from 17 histology was calculated using ssGSEA analysis from R package GSVA (24). RSEM processed and Z-score normalized RNA-Seq v2 gene expression data of TCGA cohort, downloaded from cBioPortal, was used for ssGSEA analysis. Unsupervised clustering of tumor samples was performed using hierarchical clustering with spearman correlation as the similarity metric.

Synthetic lethal partners for DDR genes in 17 cancer types are calculated using recently published DiscoverSL algorithm. For each pair of potential synthetic lethal gene pairs and a given cancer type from TCGA, DiscoverSL uses *p*-values calculated from four parameters: (a) DiffExp: differential expression of the secondary gene in samples with vs. without mutation in the primary gene (calculated from TCGA RNA-Seq raw count data available from GSE62944, using EdgeR package) (b) Exp.correlation: expression correlation of the primary and secondary gene (calculated using Pearson's correlation) (c) Mutex: mutual exclusivity of mutation of the primary and secondary gene (calculated using a hypergeometric test described in the following section), and (d) SharedPathway: probability that the primary and secondary genes are part of common pathways (Also calculated using hypergeometric test using the c2 collection from MSigDB). In DiscoverSL, these four parameters are used as features in a Random Forest model trained with a set of positive and negative examples of synthetic lethal interactions derived from literature. Detailed description for calculation of all four parameters and the Random Forest model can be found in the Supplementary Methods section of our previous publication (13).

Synthetic lethal interactions reported in previous literature or SL screens are collected from the database SynLethDB (25) and another recent publication that curated SL interactions obtained from SL screens in human cell lines (14).

### Calculation of Mutual Exclusivity and Mutual Co-occurrence

The probability of a tumor sample belonging to a DDR cluster *D* (1, 2, or 3, derived from the unsupervised clustering of ssGSEA scores of 10 DDR pathways) and co-occurrence of a gene mutation event *E* for any DDR gene (*Gene1*) associated with 10 DDR pathways is calculated with a hypergeometric test. Let *P*_*Mutco*_ be the hypergeometric *P*-values for co-occurrence of mutation in *Gene1* and DDR cluster *D*. The formula for calculation of the hypergeometric *P*-value is as follows:

$$\begin{array}{l} P_{Mutco}=\;\overset{\min\left(S_{1mut},S_{2}\right)}{\underset{{i=S}_{12mut}}{\sum }}\frac{\left(\frac{S_{1mut}}{i}\right)\left(\frac{S_{T}-S_{1mut}}{S_{2}-i}\right)}{\left(\frac{S_{T}}{S_{2}}\right)} \end{array}$$

Where,

*S*_12*mut*_ = Number of tumor samples belonging to DDR cluster D and carrying mutation in Gene1
*S*_1*mut*_ = Number of tumor samples with mutation in Gene1
*S*_2_ = Number of tumor samples belonging to DDR cluster D
*S*_*T*_ = Total Number of tumor samples

Similarly, mutual exclusivity with genetic mutation in two DDR genes Gene1 and Gene2 is calculated with a hypergeometric test that calculates the probability of co-occurrence of mutation in Gene1 and Gene2 in patient samples (from TCGA) for a given cancer. Let *P*_*Mutex*_ be the hypergeometric *P*-values for co-occurrence of mutation for Gene1 and Gene2. The formula for calculation of the hypergeometric *P*-values is as follows:

$$\begin{array}{l} P_{Mutex}=\;\overset{\min\left(S_{1mut},S_{2mut}\right)}{\underset{{i=S}_{12mut}}{\sum }}\frac{\left(\frac{S_{1mut}}{i}\right)\left(\frac{S_{T}-S_{1mut}}{S_{2mut}-i}\right)}{\left(\frac{S_{T}}{S_{2mut}}\right)} \end{array}$$

Where,

*S*_12*mut*_ = Number of cancer samples for a cancer type C with mutation in both *Gene1* and *Gene2*
*S*_1*mut*_ = Number of cancer samples for a cancer type C with mutation in *Gene1*
*S*_2*mut*_ = Number of cancer samples for a cancer type C with mutation in *Gene2*
*S*_*T*_ = Total Number of cancer samples for a cancer type C

For TCGA mutation data, cases with non-silent mutations are considered as gene mutation events. Opposite to the mutual co-occurrence, the mutual exclusivity *P*-values should represent the *P*-value for non-co-occurrence of mutations in Gene1 and Gene2. So, the mutual exclusivity *P*-value *Mutex*_*Mut*_ is calculated as:

$$\begin{array}{l} Mutex_{Mut}=1-P_{Mutex} \end{array}$$

For *Mutex*_*Mut*_, the null hypothesis is that the two genes are mutated in the same tumor samples. When *Mutex*_*Mut*_ takes a higher value (e.g., 0.98) that means the null hypothesis cannot be rejected and the gene mutations are not mutually exclusive, while *Mutex*_*Mut*_ < 0.05 means that the null hypothesis can be rejected and the gene mutations are mutually exclusive, i.e., the two genes are not mutated in the same samples.

The mutual co-occurrence and mutual exclusivity *p*-values are adjusted for multiple testing correction by false discovery rate using Benjamini and Hochberg (26).

### *In-silico* Validation of the Predicted Synthetic Lethal Interactions

We have used multiple methods to validate the significance of SL pair interactions. (1) To assess the effect of silencing the SL gene (gene2) in cancer cell lines where the primary gene (gene1) is mutated, significance of difference in shRNA score [essentiality calculated using ATARiS algorithm from shRNA screening of 214 cell lines (19)] is calculated by *t*-test using shRNA screening data from Achilles 2.4.3 project. We termed this parameter as *PvalRNAi*. (2) To assess the clinical outcome of under-expression vs. over-expression of the predicted SL gene (gene2) in cases with mutation in the primary gene (gene1), Kaplan Meier survival analysis was performed on disease free survival in TCGA clinical data. (4) To assess the potential drug sensitivity a *p*-value is calculated using *t*-test on the LNIC50 values between primary gene mutated vs. non-mutated cells from the Genomics of Drug Sensitivity in Cancer (GDSC) project data. We termed this parameter as *Drug Sensitivity*.

## Results

### Somatic Mutations in the Components of DDR in a Pan-Cancer Context

**Figure 1:**
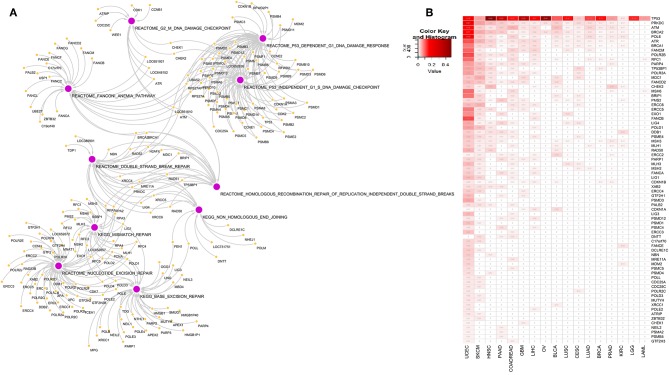
**(A)** Network diagram of genes associated with 10 DNA damage response pathways at different level of DNA repair: base excision repair, nucleotide excision repair, mismatch repair, double strand break repair, homologous recombination repair of replication independent DNA double strand breaks, non-homologous end joining, G2-M DNA damage checkpoint, Fanconi anemia pathway, P53 dependent DNA damage response and P53 independent DNA damage response. **(B)** Somatic mutations in genes from the 10 DNA damage response pathways in 17 cancer types. We included genes that are mutated in at-least 1% of tumor samples in more than one tumor types. Numbers in each cell represents frequency of samples carrying mutation in corresponding gene in that cancer type. Rows and columns are sorted by the frequency of mutation of the DDR genes in the tumor types.

To explore the association of 10 DDR pathway related alterations and gene mutations in pan-cancer context, we first identified the DDR pathway specific genes from Reactome and KEGG pathway database [MSigDB c2 collection v6.2 (18)]. These 10 pathways constitute 221 genes and represent different components of DDR handling including single strand breaks or double strand breaks in DNA as illustrated in Figure 1A and outlined in the introduction section. Next, we identified somatic mutations among the 221 genes in TCGA data comparing 17 cancer types. All cancer types had somatic alterations in one or more DDR genes. Figure 1B represents the somatic mutation in 17 TCGA cancer types. We considered genes having mutation in at least 1% samples in a given cancer, and present in any two or more cancers, resulting in 72 genes. Of all DDR genes, *TP53* was the most frequently mutated gene. The other frequently mutated DDR genes were *PRKDC, ATM, BRCA2, POLE, ATR, BRCA1*, and *FANCM*. Among the 17 cancer types, uterine corpus cancer (UCEC), head and neck cancer (HNSC), and skin cutaneous melanoma (SKCM) had most frequent alterations in DDR genes.

### Exclusive Pattern of Enrichment Is Observed Between Certain Components of DDR

**Figure 2:**
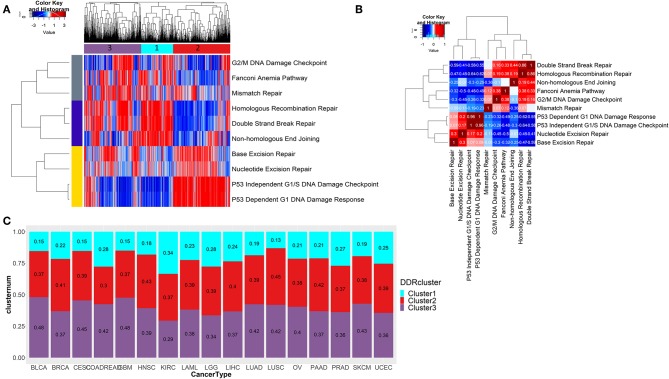
**(A)** Unsupervised clustering of ssGSEA scores of 10 DDR pathways across 7,272 tumor samples reveals three distinct clusters of tumors. A mutually exclusive enrichment pattern can be seen between these DDR pathways: P53 dependent or independent G1 DNA damage response and double strand break repair, homologous recombination repair, and non-homologous end joining. **(B)** Correlation plot showing the correlation of ssGSEA enrichments of 10 DDR pathways. **(C)** Stacked barplot shows the fraction of samples belonging to each of the three DDR clusters identified from **(A)** in each of the 17 tumor types from TCGA.

To check the difference in enrichments of the 10 DDR pathways in the pan-cancer scenario, we did single sample geneset enrichment (ssGSEA) analysis on 7,272 tumor samples from 17 tumor types from TCGA. Using unsupervised clustering, we found three pathway clusters and three sample clusters of tumor samples (Figure 2A). The sample cluster 1 had enrichment of DNA double strand break repair associated pathways, while the sample cluster 2 had enrichment in single strand break repair related pathways and *p53* dependent or independent DNA damage response at G1. Sample cluster 3, had some similar attributes from cluster 1, but can be characterized by more enrichments in G2/M cell cycle checkpoints, Fanconi anemia and mismatch repair pathways. So, the cluster 1 and cluster 3 were mostly enriched for late-stage DDR (double strand break repair and late-stage cell cycle, respectively), and cluster 1 was more enriched in early-stage DDR (single strand break repair and *p53* dependent or independent G1 checkpoint). From the correlation analysis of pan-cancer wide enrichment scores (ssGSEA) as shown in Figure 2B, we observed exclusive pattern of enrichments between the DDR pathways from three groups; one consisting of pathways related to double strand break repair (group 1; enriched in cluster 1), one consisting of pathways related to late-stage cell cycle checkpoints (G2/M), Fanconi anemia and mismatch repair (group 2; enriched in cluster 3), and the other one consisting of single strand break repair and *p53* dependent or independent DNA damage response (group 3; enriched in cluster 2). Among the pathways in group 1 and group 2, non-homologous end joining had negative correlation with the pathways in group 2, while homologous recombination had a positive correlation with these pathways. Mismatch repair had weak positive correlation with not just the pathways from the same group (Fanconi anemia and G2/M checkpoint) but also homologous recombination repair pathway from group 1. This correlation can be attributed to the fact that many components of the Fanconi anemia pathway interacts with the mismatch repair related proteins (27). The correlation of the homologous recombination repair with G2/ M DNA damage checkpoint is also expected as this pathway of double strand break repair is restricted to G2 phase or late S phase (28). Among the pathways in group 3, nucleotide and base excision repair had weak positive correlation with *p53* mediated DNA damage checkpoints. All pathways from group 3 had negative correlations with the pathways from group 1 and group 2. *P53* dependent or independent DNA damage response showed strongest negative correlation with homologous recombination repair (*r* < −0.6). From published literature we see that *p53* has direct role in suppressing homologous recombination repair of DNA double strand breaks (29). Thus, tumors may undergo DNA double strand break repair through activated homologous recombination repair in the absence of *p53* mediated apoptosis, while tumors are most likely to undergo cell cycle arrest at G1 phase by intervention of *p53*. We checked the distribution the samples assigned to these three clusters in each of the 17 cancer types (shown in Figure 2C). Though there was some variability in distribution of three clusters in different cancer types, cluster 1 had the lowest frequency of samples in almost all cancer types (only kidney renal cell carcinoma KIRC had almost equal frequency of all 3 clusters).

### Underlying Gene Mutation Signatures of the Three Major DDR Clusters

**Figure 3:**
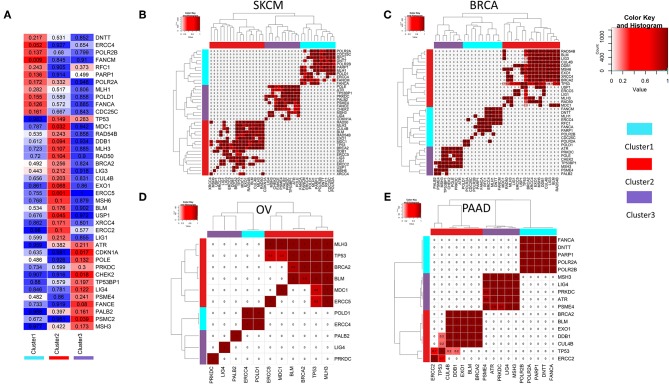
**(A)** Mutual co-occurrence of certain DDR genes was observed with each of the three DDR clusters identified in 2b. In the matrix, the color key blue to red denotes the tendency from mutual exclusivity to mutual co-occurrence of the corresponding gene mutation (y-axis) and the corresponding DDR cluster (x-axis). The *p*-values for co-occurrence of DDR gene mutations and DDR clusters calculated using hypergeometric test are shown in each cell. **(B–E)** Mutually exclusive mutations of DDR genes representing three different DDR clusters (from 3a) is shown for tumor types **(B)** SKCM **(C)** BRCA **(D)** OV **(E)** PAAD.

To compare the somatic mutations among DDR pathway genes between the 3 clusters (obtained from the ssGSEA analysis, see Figure 2A), we performed a statistical test for mutual co-occurrence of the somatic mutations of the 221 genes among the three DDR clusters. Figure 3A, represents 40 genes (hypergeometric test, FDR corrected *p* < 0.3) showing higher occurrence of somatic mutations. These genes formed mutually exclusive pattern between the DDR clusters. The mutated genes representing cluster 1 were mostly associated with nucleotide excision repair (*POLR2A, POLR2B, ERCC4, POLD1*), mismatch repair (*RFC1, MLH1*), base excision repair (*POLD1, PARP1*), and Fanconi anemia (*FANCM, FANCA*). The mutated genes in tumors from cluster 2 were mostly associated with homologous recombination repair (*BRCA2, RAD50, RAD54B, BLM, MDC1, LIG1*), non-homologous end joining (*RAD50, XRCC4*), *ATM* pathway (*TP53*), Fanconi anemia (*BRCA2, USP1*), meiotic recombination (*MLH3, RAD50, BLM*) and also mismatch repair (*EXO1, MSH6, MLH3*), base excision repair (*LIG1, LIG3*), and nucleotide excision repair (*ERCC5, DDB1, ERCC2, CUL4B, LIG1*). The genes mutated in cluster 3 were mainly associated with cell cycle checkpoints (*CHEK2, ATR, CDKN1A, POLE, PSME4, PSMC2, PRKDC*), and additionally with Fanconi anemia (*FANCE, PALB2*), and non-homologous end joining (*LIG4, PRKDC*). Moreover, in all cancer types, we observed mutually exclusive pattern of mutations between the clusters, but not within the same cluster (Figures 3B–E, Supplementary Figure 1; *p*-value calculated using hypergeometric test followed by FDR correction).

### Analysis of Transcriptome-Wide Synthetic Lethal Candidates Identifies Common and Exclusive Targets for Different DDR Clusters

**Figure 4:**
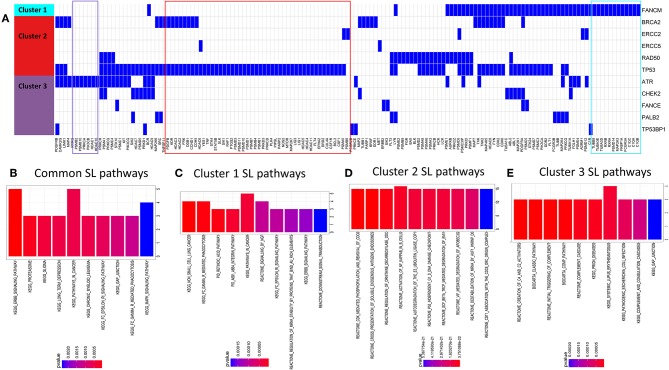
**(A)** Predicted SL interactors of genes mutated in cluster 1, cluster 2, or cluster 3. Blue denotes an SL interaction and white denotes no SL interaction between the corresponding SL gene (columns) and DDR cluster (row). We can see there is some overlap and some exclusivity between SL partners for DDR genes from different DDR clusters. **(B–E)** Barcharts show pathways enriched for predicted synthetic lethal partners **(B)** common for all clusters **(C–E)**, unique for gene mutations specific to DDR clusters 1, 2 and 3, respectively.

We looked for potential synthetic lethal partners of the cluster specific 40 significant DDR genes (see Figure 3A) using two approaches: (1) from published synthetic lethal screens in human cell lines (14, 25) and (2) using our previously published machine-learning based algorithm DiscoverSL (13). To shortlist the most probable SL candidates from the DiscoverSL predictions, we applied two *in-silico* validation approach. First, we calculated the conditional essentiality of the SL interaction, i.e., the statistical significance of difference between the shRNA scores (targeting the synthetic lethal gene) for human cell lines with or without mutation in the primary gene; and second, we performed Kaplan-Meier analysis on TCGA clinical data to check if the primary gene is mutated, the differences in disease-free survival between patients when the SL interactor gene downregulated (expression < median) compared to samples where the gene is upregulated (expression > median). The SL pairs which showed significant effects of co-inactivation from both validation methods were chosen as the final list of most probable SL interactors for DDR genes. Figure 4A shows a representation of selected SL interactors for genes from the three DDR clusters. A subset of genes was shown to be exclusively associated with each cluster (shown as colored boxes in Figure 4A). We checked the functional enrichments of the common and exclusive SL interactors of these DDR genes. The common SL interactors for all three clusters were enriched for *MAPK* pathway, *ERBB* pathway, *GAP* junction, and proteasomes (Figure 4B). Functional enrichment analysis exclusive to the three DDR clusters (Figures 4C–E) showed that, cluster 1 is associated with *NGF* signaling, *ERBB* signaling, integrin pathway, retinoic acid pathway, Fc gamma mediated phagocytosis; cluster 2 is found to be enriched with cell cycle and immune system associated pathways were enriched, and cluster 3 had mostly immune response related pathways.

### DDR Genes Having Distinct Alteration Patterns Between Different DDR Clusters Are Potentially Synthetic Lethal

**Figure 5:**
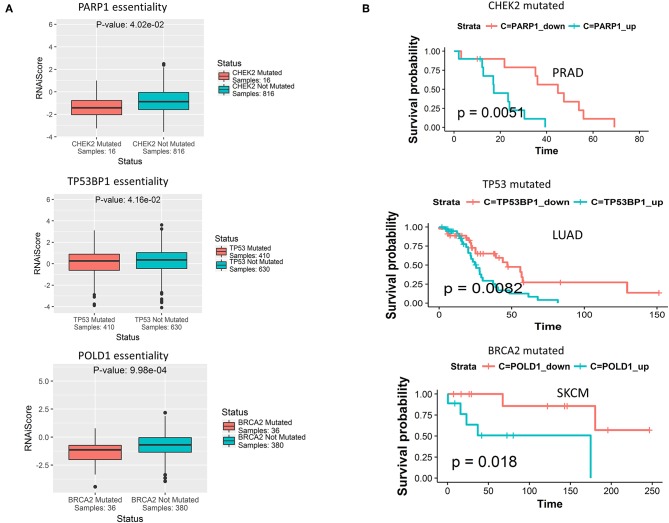
**(A)** Predicted synthetic lethal partners for gene mutations specific to each DDR cluster, that have conditional essentiality in presence of the corresponding gene mutations as observed from cancer cell line RNAi screening data from Achilles portal. The term RNAiScore in y-axis is used to represent the essentiality score of genes in shRNA screenings processed by ATARiS algorithm, as collected from the Achilles project v 2.4.3 (described in the data collection section in Methods). The more negative RNAiScore, the more essential the corresponding gene. **(B)** From TCGA genomic and clinical data, certain predicted synthetic lethal genes of the primary genes from each of the three DDR cluster show survival advantage in terms of increased disease-free survival when down-regulated compared to when up-regulated (down<median, up>median) in certain cancer types in presence of somatic mutations in that DDR gene.

Having observed mutually exclusive mutation pattern of 40 DDR genes associated with different clusters (shown in selected cancers in Figures 3B–E), we wanted to see if these genes also exhibit synthetic lethality. To test this hypothesis, we searched for synthetic lethal interactions between DDR genes from published synthetic lethal screens in human cell lines. We identified SL interactions between multiple DDR genes that supported our postulation (Supplementary Table 1). Further, from the predicted SL interactions from DiscoverSL we found many potential SL relationships that fulfilled the 2 *in silico* validation criteria mentioned above (Supplementary Table 1). A representation of 3 SL pairs one from each cluster, that passed our rigorous filter criteria were shown in Figure 5. The filtered SL pairs are validated *in-silico* at shRNA level (Figure 5A), and by Kaplan Meier analysis showing the clinical relevance of the same pairs as disease-free survival when ± mutation in one gene had a significant association of over (>median) or under (< median) expression of SL partner gene (Figure 5B). As shown from the figure, *PARP1* (cluster 1) was found to have conditional essentiality with cell lines having mutation in *CHEK2* (cluster 3) and a survival advantage of down regulation of *PARP1* in prostate cancer patients when *CHEK2* was mutated. Similarly, *TP53BP1* (cluster 3) has conditional essentiality in cell lines having mutation in *TP53* (cluster 2) with survival advantage in lung cancer patients, and *POLD1* (cluster 3) has conditional essentiality in cell lines having mutation in *BRCA2* (cluster 1) and survival advantage shown in skin cancer patients. A complete list of SL interactions (previously reported or novel prediction) is shown in Supplementary Table 1.

### Analysis of Drug Sensitivity Associated With Mutations in DDR Genes From Different Clusters

**Figure 6:**
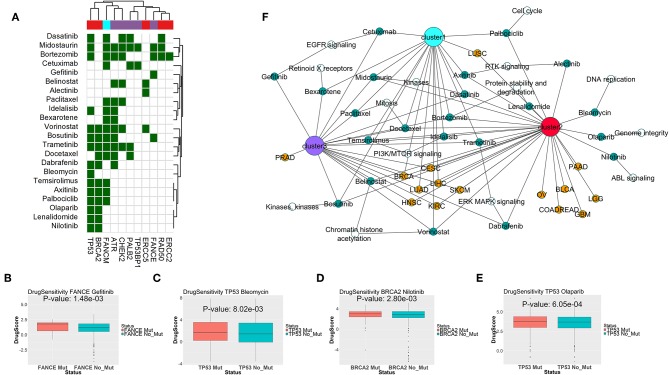
**(A)** Drugs targeting predicted synthetic lethal partners for gene mutations specific to each DDR cluster, that have conditional sensitivity in presence of the corresponding gene mutations as observed from cancer cell line drug screening data from GDSC portal. In the matrix, color coding of each cell denotes whether the corresponding drug (in y-axis) is sensitive to mutations in the corresponding DDR gene (in x-axis). The column color labels annotate the DDR clusters where mutations in the corresponding DDR gene is prevalent. **(B–E)** Sensitivities of Gefitinib in presence of FANCE mutation, Bleomycin in presence of TP53 mutation, Nilotinib in presence of BRCA2 mutation, and Olaparib in presence of TP53 mutation; as seen from cancer cell lines in GDSC portal. The DrugScore in the y-axis represents LN IC50 values of the drugs in the cell lines with/without mutation in the corresponding DDR genes. **(F)** Association network of predicted synthetic lethal genes of gene mutations specific to each DDR cluster across TCGA tumor types, and potential drugs targeting the synthetic lethal genes which are also sensitive to mutations in the primary DDR gene. The following color coding is applied; tumor types: red, DDR gene mutations specific to DDR cluster 1: cyan, DDR gene mutations specific to DDR cluster 2: pink, DDR gene mutations specific to DDR cluster 3: purple, synthetic lethal genes: white and drugs: green.

To find potential drugs for targeting the SL interactors of DDR genes from different clusters, we combined the drug-target information from the databases DrugBank (21) and DGIdb (22), and the drug sensitivity data in cell lines from GDSC portal (23). We limited our drug search to only the drugs approved by FDA for treating cancers, as per the National Cancer Institute resource (https://www.cancer.gov/about-cancer/treatment/drugs). For the drugs targeting SL interactors of the DDR genes from each cluster, we calculated the relative drug sensitivity in presence of mutations in the primary gene. Figure 6A shows the drugs targeting potential SL interactors for the mutations in the primary DDR genes from different clusters. Drugs showing significantly increased sensitivity for specific DDR gene mutations highlighted with green (*p* < 0.1, one-sided *t*-test). Combining the drug sensitivity results with the information on the potential SL interactions from literature and computer predictions, we generated a network of the DDR gene alterations, SL interactions and drugs (Figure 6B). The SL interactions are restricted to only those showing significant clinical benefit from the disease-free survival analysis (described in the previous section). The drug Gefitinib (targeting *EGFR* signaling) was only seen to have sensitivity for the gene *FANCE* from DDR cluster 3 (Figure 6C). The drug Bleomycin (targeting DNA replication, Figure 6D) showed sensitivity to only TP53 mutation. The drugs Olaparib (*PARP*-inhibitor, Figure 6E), Nilotinib (targeting *ABL* signaling, Figure 6F), Lenalidomide targeting protein stability, and Alectinib targeting *RTK* signaling was only seen to have sensitivity for genes from DDR cluster 2 (*BRCA2, TP53*, or *ERCC5*). The drugs Vorinostat, Trametinib, Idelalisib, Docetaxel, Bortezomib, Dasatinib, and Midostaurin, targeting histone acetylation, *PI3K/MTOR* signaling, *ERK/MAPK* signaling, mitosis, proteasome and kinases, respectively, showed sensitivity for DDR genes from all three clusters (Figures 6A,F).

## Discussion

DNA damage response alterations are vital to the transformed cells to evade senescence. But at the same time, these alterations which are common in cancers are also supposed as “Achilles heel” of the cancer that makes them vulnerable to certain cytotoxic or targeted therapies (30). In order to get an understanding of potential targets specific to different DDR alterations, we performed an analysis of multi-cancer study on the patterns of alteration in 10 DDR pathways across 7,272 tumors from 17 tumor histology in TCGA. We identified distinct sample clusters based on defect in DDR mechanism rather than by histology. This pattern of exclusive enrichment of certain DDR pathways and depletion of others is expected as tumors with defects in certain DDR pathways tend to rely on the residual DDR pathways to evade apoptosis resulting from genotoxic stress (7). Cancer type-specific distribution of the number of tumor samples belonging to these three clusters showed that all cancer types had a higher fraction belonging to defects in double strand break repair pathways, such as homologous recombination, which is consistent with the observations from a previous pan-cancer study (6).

Notably, looking at the underlying genomic signatures of the DDR clusters, we found that the somatic mutation patterns of genes from different clusters showed a clear mutually exclusive signature in all cancers (see Figure 3A). Association of the genes representing the three clusters indicates that these genes were involved in complementary DDR pathways; cluster 1 has genetic alterations related to single strand break repair pathways like base excision and nucleotide excision repair. Cluster 2 has genetic alterations related to homologous recombination, non-homologous end joining repair, and nucleotide excision repair. Cluster 3 has genetic alterations mostly related to cell cycle checkpoints. In support of our findings, a considerable crosstalk among the single- and double-strand lesion repair pathways and replication fork restart pathways has been reported by several studies. A functional crosstalk was shown in which overexpression of a DNA repair component in one pathway compensates for a repair defect in another, conferring therapeutic resistance (31). A signaling crosstalk between the homologous recombination and canonical non-homologous end joining pathways through *ATR, ATM*, and *DNA-PK* has been reported (32, 33). Finally, a direct crosstalk when specific components are shared among pathways, for example, *PARP1* functions in base excision repair and in alternative non-homologous end joining (34). These findings suggest that simultaneous alterations in these pathways will be potentially detrimental to the tumor cells. Identifying cancers that are functionally defective in specific repair pathways could benefit DNA-repair targeted therapies (35). From our findings, we showed that there are indeed potential SL relationships between genes from different clusters, and their co-inactivation can be lethal to the tumor cells (as seen in Figure 5, from RNAi screening data, e.g., *CHEK2* and *PARP1, TP53* and *TP53BP1, BRCA2* and *POLD1*). As many of the SL interactions found from the essentiality screens do not translate into clinically beneficial targets, we also checked from TCGA clinical data, whether co-inactivation of the potential SL candidates show a significant increase in disease-free survival time. Among the potential SL interactors from different clusters, *BRCA2* (cluster 2) and *PARP1* (cluster 1) have been reported in literature as SL partners (26). Additionally, we found potential SL relationships between *TP53* (cluster 2) and *TP53BP1* (cluster 3), and *CHEK2* (cluster 3) with *PARP1* (cluster 1) that has not been reported previously but show clinical benefits upon co-inactivation in lung adenocarcinoma (LUAD) and prostate adenocarcinoma (PRAD), respectively. We observed that co-inactivation of potential SL partners does not always show significant clinical benefit (in increased survival time) in all tumor types. The varying sensitivity to co-inactivation of same SL partners in different tumor types may be linked to the underlying heterogeneity of different tumor histology.

Given the importance of DDR pathways in cancer, we hypothesize the occurrence of common SL mechanisms between cancer types. We identified SL genes common to all clusters, that are associated with *MAPK, ERBB*, proteasome pathways. From the drug sensitivity data, the cancer drugs targeting kinases (Dasatinib, Bosutinib, Axitinib, Alectinib), *PI3K/MTOR* pathway (Idelalisib, Temsirolimus), *MEK* pathway (Trametinib, Dabrafenib), *EGFR* signaling (Cetuximab, Gefitinib), proteasome (Bortezomib), *HDAC*s (Vorinostat, Belinostat), cell cycle (Paclitaxel, Docetaxel, Palbociclib), Retinoid receptors (Bexarotene) were found to be sensitive to mutations in genes from multiple DDR clusters. The importance of the receptor tyrosine kinase signaling (*EGFR/MEK/ERK/PI3K*) in regulation of DDR pathways and mediating radiation or chemo resistance is well-known, and many ongoing clinical trials are investigating the potential of combination therapies involving DDR inhibitors and tyrosine kinase inhibitors in cancers [reviewed by (36)]. Also, there is evidence of *HDAC* inhibitors triggering DNA damage in cancer cells which further attenuates by DNA-damaging chemotherapy or radiation (37). So, inactivation of DDR proteins may sensitize cancer cells to *HDAC* inhibitors, as we see from our analysis. There are reports connecting proteasomes to DDR pathways and proteasome inhibitors are shown to enhance sensitization of cancer cells to DNA damaging agents (38). Consistently, our analysis indicates that co-inactivation of DDR genes combined with proteasome inhibitors may be lethal to cancer cells.

Among the SL interactor pathways exclusive to DDR cluster 1, there were pathways associated with efficient DNA double strand break repair, e.g., integrin, *ERBB* pathways. It has been previously shown that activated *ERBB* pathway can trigger DNA double strand break repair (39) and disabling the *ERBB* pathway resulted in genotoxic cell death induced by radiation (40). Similarly, it has been shown that beta integrins can positively regulate components of homologous recombination repair of DNA double strand breaks, facilitating resistance to radiation-induced cell death (41). Thus, co-inactivation of single strand break repair (which is predominantly associated with DDR cluster 1) with *ERBB* or integrin pathway can be lethal to cancer cells. This observation is further supported by our drug sensitivity analysis, as we observed sensitivity of *EGFR* signaling inhibitor drug cetuximab to be sensitive to alterations in cluster 1 (Figure 6F).

The SL interaction of cluster 2 with cell cycle related pathways was also expected from our analysis, as the gene alterations specific to cluster 3 were mostly associated with cell cycle checkpoints. Consistently, from the drug sensitivity analysis, we found sensitivity of the drug Palbociclib (targets cell cycle) in presence of alterations in cluster 2. Besides them, some drugs were only sensitive to mutations in cluster 2 which was mostly associated with double strand break repair; e.g., Olaparib (*PARP* inhibitor), Nilotinib (*ABL* inhibitor), and Bleomycin (DNA ligase inhibitor). *PARP* inhibitor drugs are the first ever FDA-approved therapies for treating tumors deficient in homologous recombination repair (42). In case of Bleomycin, we found literature reports supporting the sensitivity to Bleomycin by impairing *p53* function in transgenic mice (43).

Interestingly, the SL interactors of gene alterations specific to cluster 3 were mostly enriched for immune system mediated cell killing, e.g., complement cascade associated with innate immunity. As stated earlier, the DDR cluster 3 was mostly associated with alterations in cell cycle checkpoint genes, e.g., *CHEK2, ATR, PRKDC*. It was reported that inhibition of cell cycle components (*CDK4/6*) can trigger anti-tumor immune response (44). Also, DDR signaling is involved in innate immune response, and currently DDR inhibitors (*ATR* or *PARP1* inhibitors) combined with immune checkpoint inhibitors are undergoing clinical trials (45–47).

In summary, DNA and DNA damage response proteins have incredible potential as next generation therapeutic targets for the treatment of multiple cancers. While long term effects of DDR inhibition have yet to be understood in patients, and the potential for the emergence of secondary cancers exists, there is significant evidence at both the preclinical and early clinical stage that this specific targeting strategy will be the next breakthrough in cancer therapy. Our systematic analysis of multi-cancer SL targets and drug sensitivity revealed many potential drug targets for treating cancers deficient in DNA damage response in addition to *PARP* inhibitors and established a framework to explore and prioritize the potential targeted therapies for certain DDR alterations in cancer.

## Data Availability Statement

The datasets generated for this study can be found in the http://cancergenome.nih.gov/abouttcga/overview/howitworks/datasharingmanagement.

## Author Contributions

SD and US conceived and designed the study. SD developed the computational algorithms and implementations under the supervision of KC and US. Result interpretations done by SD, US, and KC.

### Conflict of Interest

The authors declare that the research was conducted in the absence of any commercial or financial relationships that could be construed as a potential conflict of interest.

## Supplementary Material

Supplementary Figure 1 Mutually exclusive mutations of DDR genes representing three different DDR clusters (from Figure 3A) is shown for 15 tumor types.

Supplementary Table 1 This table contains a filtered list of the literature reported and predicted synthetic lethal interactions of DDR genes from 3 clusters in TCGA tumor types. The synthetic lethal interactions presented in this table has the following filters: (1) the primary gene (Gene1) has mutation in at-least 5 tumor samples from the corresponding TCGA tumor type (2) the primary gene (Gene1) and the synthetic lethal gene (Gene2) has significant conditional essentiality from the Achilles shRNA screening data (*p* < 0.1) (3) there is significant difference in survival between patients having high or low expression of the synthetic lethal gene (Gene2) in presence of mutation in Gene1 (*p* < 0.1) (4) there is significant difference in drug sensitivity for drugs targeting Gene2 in cell lines with or without mutation in Gene1 (*p* < 0.1).

## References

[1] d'Adda di Fagagna F Reaper PM Clay-Farrace L Fiegler H Carr P Von Zglinicki T. A DNA damage checkpoint response in telomere-initiated senescence. Nature. (2003) 426:194–8. 10.1038/nature02118 14608368

[2] Jackson SP Bartek J. The DNA-damage response in human biology and disease. Nature. (2009) 461:1071–8. 10.1038/nature08467 19847258 PMC2906700

[3] Negrini S Gorgoulis VG Halazonetis TD Genomic instability–an evolving hallmark of cancer. Nat Rev Mol Cell Biol. (2010) 11:220–8. 10.1038/nrm2858 20177397

[4] Goldstein M Kastan MB. The DNA damage response: implications for tumor responses to radiation and chemotherapy. Annu Rev Med. (2015) 66:129–43. 10.1146/annurev-med-081313-121208 25423595

[5] Hoeijmakers JH. Genome maintenance mechanisms for preventing cancer. Nature. (2001) 411:366–74. 10.1038/35077232 11357144

[6] Knijnenburg TA Wang L Zimmermann MT Chambwe N Gao GF Cherniack AD. Genomic and molecular landscape of DNA damage repair deficiency across the cancer genome atlas. Cell Rep. (2018) 23:239–54.e6. 10.1016/j.celrep.2018.03.076 29617664 PMC5961503

[7] Dietlein F Thelen L Reinhardt HC. Cancer-specific defects in DNA repair pathways as targets for personalized therapeutic approaches. Trends Genet. (2014) 30:326–39. 10.1016/j.tig.2014.06.003 25017190

[8] Lord CJ Ashworth A. PARP inhibitors: synthetic lethality in the clinic. Science. (2017) 355:1152–8. 10.1126/science.aam7344 28302823 PMC6175050

[9] Luo J Emanuele MJ Li D Creighton CJ Schlabach MR Westbrook TF. A genome-wide RNAi screen identifies multiple synthetic lethal interactions with the Ras oncogene. Cell. (2009) 137:835–48. 10.1016/j.cell.2009.05.006 19490893 PMC2768667

[10] Turner NC Lord CJ Iorns E Brough R Swift S Elliott R. A synthetic lethal siRNA screen identifying genes mediating sensitivity to a PARP inhibitor. EMBO J. (2008) 27:1368–77. 10.1038/emboj.2008.61 18388863 PMC2374839

[11] Jerby-Arnon L Pfetzer N Waldman YY McGarry L James D Shanks E. Predicting cancer-specific vulnerability via data-driven detection of synthetic lethality. Cell. (2014) 158:1199–209. 10.1016/j.cell.2014.07.027 25171417

[12] Lu X Megchelenbrink W Notebaart RA Huynen MA. Predicting human genetic interactions from cancer genome evolution. PLoS ONE. (2015) 10:e0125795. 10.1371/journal.pone.0125795 25933428 PMC4416779

[13] Das S Deng X Camphausen K Shankavaram U. DiscoverSL: an R package for multi-omic data driven prediction of synthetic lethality in cancers. Bioinformatics. (2019) 35:701–2. 10.1093/bioinformatics/bty673 30059974 PMC6378931

[14] Lee JS Das A Jerby-Arnon L Arafeh R Auslander N Davidson M. Harnessing synthetic lethality to predict the response to cancer treatment. Nat Commun. (2018) 9:2546. 10.1038/s41467-018-04647-1 29959327 PMC6026173

[15] Cancer Genome Atlas Research Weinstein JN Collisson EA Mills GB Shaw RMK Ozenberger BA. The Cancer Genome Atlas Pan-Cancer analysis project. Nat Genet. (2013) 45:1113–20. 10.1038/ng.2764 24071849 PMC3919969

[16] Cerami E Gao J Dogrusoz U Gross BE Sumer SO Aksoy BA. The cBio cancer genomics portal: an open platform for exploring multidimensional cancer genomics data. Cancer Discov. (2012) 2:401–4. 10.1158/2159-8290.CD-12-0095 22588877 PMC3956037

[17] Rahman M Jackson LK Johnson WE Li DY Bild AH Piccolo SR. Alternative preprocessing of RNA-Sequencing data in The Cancer Genome Atlas leads to improved analysis results. Bioinformatics. (2015) 31:3666–72. 10.1093/bioinformatics/btv377 26209429 PMC4804769

[18] Liberzon A Subramanian A Pinchback R Thorvaldsdottir H Tamayo P Mesirov JP. Molecular signatures database (MSigDB) 3.0. Bioinformatics. (2011) 27:1739–40. 10.1093/bioinformatics/btr260 21546393 PMC3106198

[19] Cowley GS Weir BA Vazquez F Tamayo P Scott JA Rusin S. Parallel genome-scale loss of function screens in 216 cancer cell lines for the identification of context-specific genetic dependencies. Sci Data. (2014) 1:140035. 10.1038/sdata.2014.35 25984343 PMC4432652

[20] Barretina J Caponigro G Stransky N Venkatesan K Margolin AA Kim S. The Cancer Cell Line Encyclopedia enables predictive modelling of anticancer drug sensitivity. Nature. (2012) 483:603–7. 10.1038/nature11003 22460905 PMC3320027

[21] Wishart DS Feunang YD Guo AC Lo EJ Marcu A Grant JR. DrugBank 5.0: a major update to the DrugBank database for 2018. Nucleic Acids Res. (2018) 46:D1074–82. 10.1093/nar/gkx1037 29126136 PMC5753335

[22] Cotto KC Wagner AH Feng YY Kiwala S Coffman AC Spies G. DGIdb 3.0: a redesign and expansion of the drug-gene interaction database. Nucleic Acids Res. (2018) 46:D1068–73. 10.1093/nar/gkx1143 29156001 PMC5888642

[23] Yang W Soares J Greninger P Edelman EJ Lightfoot H Forbes S. Genomics of Drug Sensitivity in Cancer (GDSC): a resource for therapeutic biomarker discovery in cancer cells. Nucleic Acids Res. (2013) 41:D955–61. 10.1093/nar/gks1111 23180760 PMC3531057

[24] Hanzelmann S Castelo R Guinney J. GSVA: gene set variation analysis for microarray and RNA-seq data. BMC Bioinformatics. (2013) 14:7. 10.1186/1471-2105-14-7 23323831 PMC3618321

[25] Guo J Liu H Zheng J. SynLethDB: synthetic lethality database toward discovery of selective and sensitive anticancer drug targets. Nucleic Acids Res. (2016) 44:D1011–7. 10.1093/nar/gkv1108 26516187 PMC4702809

[26] Benjamini Y Hochberg Y Controlling the false discovery rate: a practical and powerful approach to multiple testing. J R Stat Soc. (1995) 57:289–300. 10.1111/j.2517-6161.1995.tb02031.x

[27] Peng M Xie J Ucher A Stavnezer J Cantor SB. Crosstalk between BRCA-Fanconi anemia and mismatch repair pathways prevents MSH2-dependent aberrant DNA damage responses. Embo J. (2014) 33:1698–712. 10.15252/embj.201387530 24966277 PMC4194102

[28] Moynahan ME Jasin M. Mitotic homologous recombination maintains genomic stability and suppresses tumorigenesis. Nat Rev Mol Cell Biol. (2010) 11:196–207. 10.1038/nrm2851 20177395 PMC3261768

[29] Linke SP Sengupta S Khabie N Jeffries BA Buchhop S Miska S. p53 interacts with hRAD51 and hRAD54, and directly modulates homologous recombination. Cancer Res. (2003) 63:2596–605. 12750285

[30] O'Connor MJ. Targeting the DNA Damage Response in Cancer. Mol Cell. (2015) 60:547–60. 10.1016/j.molcel.2015.10.040 26590714

[31] Kelley MR Logsdon D Fishel ML. Targeting DNA repair pathways for cancer treatment: what's new? Future Oncol. (2014) 10:1215–37. 10.2217/fon.14.60 24947262 PMC4125008

[32] Ashley AK Shrivastav M Nie J Amerin C Troksa K Glanzer JG. DNA-PK phosphorylation of RPA32 Ser4/Ser8 regulates replication stress checkpoint activation, fork restart, homologous recombination and mitotic catastrophe. DNA Repair. (2014) 21:131–9. 10.1016/j.dnarep.2014.04.008 24819595 PMC4135522

[33] Liu S Opiyo SO Manthey K Glanzer JG Ashley AK Amerin C. Distinct roles for DNA-PK, ATM and ATR in RPA phosphorylation and checkpoint activation in response to replication stress. Nucleic Acids Res. (2012) 40:10780–94. 10.1093/nar/gks849 22977173 PMC3510507

[34] Wang M Wu W Wu W Rosidi B Zhang L Wang H. PARP-1 and Ku compete for repair of DNA double strand breaks by distinct NHEJ pathways. Nucleic Acids Res. (2006) 34:6170–82. 10.1093/nar/gkl840 17088286 PMC1693894

[35] Mirza MR Monk BJ Herrstedt J Oza AM Mahner S Redondo A. Niraparib maintenance therapy in platinum-sensitive, recurrent ovarian cancer. N Engl J Med. (2016) 375:2154–64. 10.1056/NEJMoa1611310 27717299

[36] Mahajan K Mahajan NP. Cross talk of tyrosine kinases with the DNA damage signaling pathways. Nucleic Acids Res. (2015) 43:10588–601. 10.1093/nar/gkv1166 26546517 PMC4678820

[37] Robert C Rassool FV. HDAC inhibitors: roles of DNA damage and repair. Adv Cancer Res. (2012) 116:87–129. 10.1016/B978-0-12-394387-3.00003-3 23088869

[38] Jacquemont C Taniguchi T. Proteasome function is required for DNA damage response and fanconi anemia pathway activation. Cancer Res. (2007) 67:7395–405. 10.1158/0008-5472.CAN-07-1015 17671210

[39] Mukherjee B McEllin B Camacho CV Tomimatsu N Sirasanagandala S Nannepaga S. EGFRvIII and DNA double-strand break repair: a molecular mechanism for radioresistance in glioblastoma. Cancer Res. (2009) 69:4252–9. 10.1158/0008-5472.CAN-08-4853 19435898 PMC2694953

[40] O'Rourke DM Kao GD Singh N Park BW Muschel RJ Wu CJ. Conversion of a radioresistant phenotype to a more sensitive one by disabling erbB receptor signaling in human cancer cells. Proc Natl Acad Sci USA. (1998) 95:10842–7. 10.1073/pnas.95.18.10842 9724792 PMC27983

[41] Ahmed KM Pandita RK Singh DK Hunt CR Pandita TK. beta1-integrin impacts Rad51 Stability and DNA double-strand break repair by homologous recombination. Mol Cell Biol. (2018) 38:e00672–17. 10.1128/MCB.00672-17 29463647 PMC5902589

[42] Farmer H McCabe N Lord CJ Tutt NJA Johnson DA Richardson TB. Targeting the DNA repair defect in BRCA mutant cells as a therapeutic strategy. Nature. (2005) 434:917–21. 10.1038/nature03445 15829967

[43] Ghosh S Mendoza T Ortiz LA Hoyle GW Fermin CD Brody AR. Bleomycin sensitivity of mice expressing dominant-negative p53 in the lung epithelium. Am J Respir Crit Care Med. (2002) 166:890–7. 10.1164/rccm.2109094 12231503

[44] Chaikovsky AC Sage J. Beyond the cell cycle: enhancing the immune surveillance of tumors via CDK4/6 inhibition. Mol Cancer Res. (2018) 16:1454–7. 10.1158/1541-7786.MCR-18-0201 29934327 PMC6170710

[45] Higuchi T Flies DB Marjon NA Mantia-Smaldone G Ronner L Gimotty PA. CTLA-4 blockade synergizes therapeutically with PARP inhibition in BRCA1-deficient ovarian cancer. Cancer Immunol Res. (2015) 3:1257–68. 10.1158/2326-6066.CIR-15-0044 26138335 PMC4984269

[46] Sato H Niimi A Yasuhara T Permata BMT Hagiwara Y Isono M. DNA double-strand break repair pathway regulates PD-L1 expression in cancer cells. Nat Commun. (2017) 8:1751. 10.1038/s41467-017-01883-9 29170499 PMC5701012

[47] Le DT Uram JN Wang H Bartlett BR Kemberling H Eyring AD. PD-1 blockade in tumors with mismatch-repair deficiency. N Engl J Med. (2015) 372:2509–20. 10.1056/NEJMoa1500596 26028255 PMC4481136

