# Supplementary figures and images for: Pan-Cancer Analysis of Potential Synthetic Lethal Drug Targets Specific to Alterations in DNA Damage Response
Source: Front Oncol. 2019 Oct 25;9:1136. doi: 10.3389/fonc.2019.01136 (PMC6823874; doi:10.3389/fonc.2019.01136)

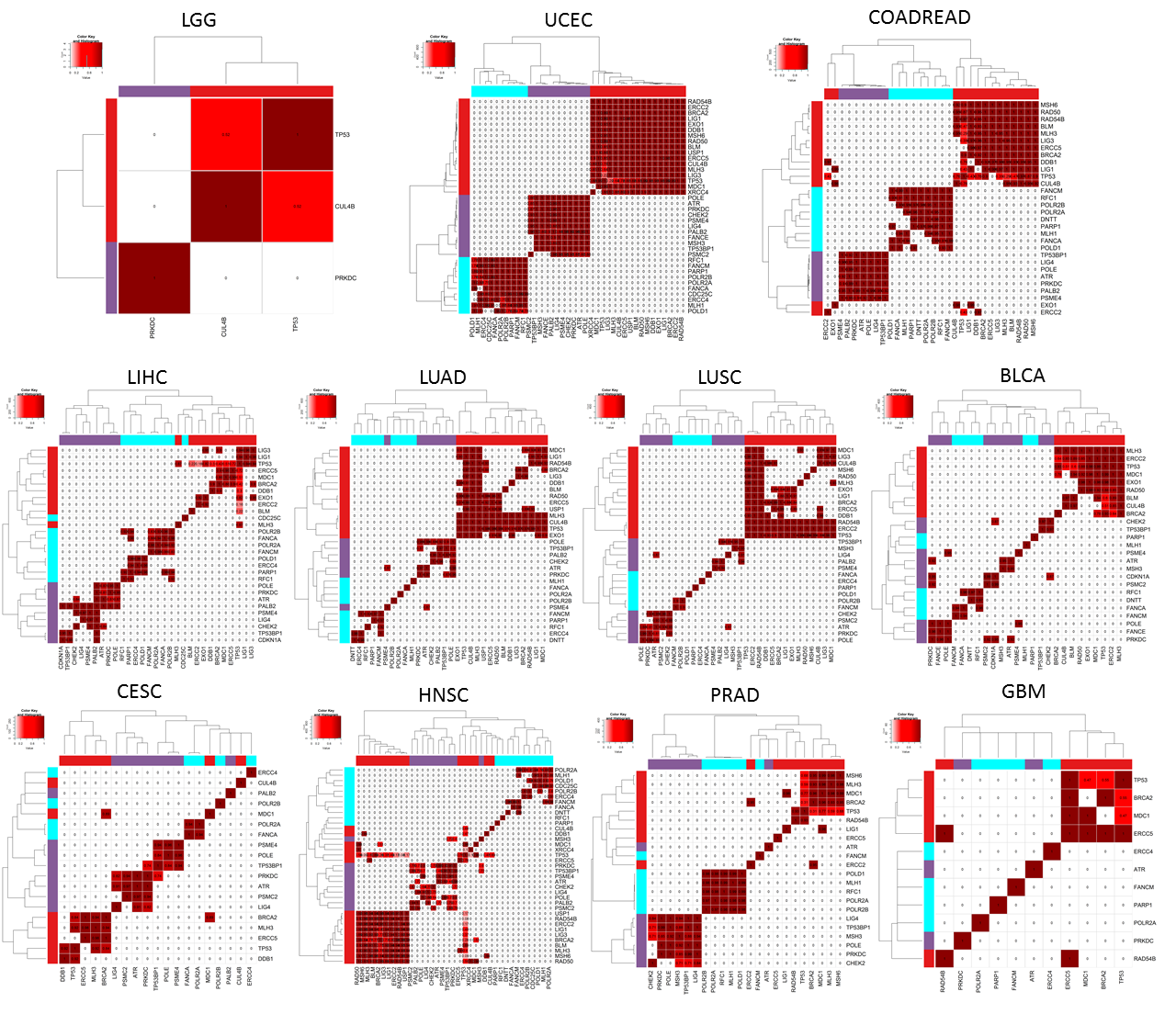

Supplement: Supplementary Figure 1 — Mutually exclusive mutations of DDR genes representing three different DDR clusters (from Figure 3A) is shown for 15 tumor types. [file Image_1.TIF]
